# Cholesterol: An Important Determinant of Muscle Atrophy in Astronauts

**DOI:** 10.26502/jbb.2642-91280072

**Published:** 2023-03-02

**Authors:** Hoangvi Le, Vikrant Rai, Devendra K Agrawal

**Affiliations:** Department of Translational Research, Western University of Health Sciences, Pomona, California 91766, USA

**Keywords:** Astronauts, Cholesterol, Hypercholesterolemia, Microgravity, Muscle atrophy, Space flight

## Abstract

Since cholesterol is not routinely measured in astronauts before and after their return from space, there is no data on the role of blood cholesterol level in muscle atrophy and microgravity. Since the first moon landing, aerospace medicine became outdated and has not pushed boundaries like its rocket engineering counterpart. Since the 2019 astronaut twin study, there has yet to be another scientific breakthrough for aerospace medicine. Microgravity-induced muscle atrophy is the most known consequence of spaceflight. Yet, so far, there is no therapeutic solution to prevent it or any real efforts in understanding it on a cellular or molecular level. The most obvious reason to this unprecedented level of research is due to the small cohort of astronauts. With the establishment of private space industries and exponential recruitment of astronauts, there is more reason to push forward spaceflight-related health guidelines and ensure the safety of the brave humans who risk their lives for the progression of mankind. Spaceflight is considered the most challenging job and the failure to prevent injury or harm should be considered reckless negligence by the institutions that actively prevented sophistication of aerospace medicine. In this critical review, role of cholesterol is analyzed across the NASA-established parameters of microgravity-induced muscle atrophy with a focus on potential therapeutic targets for research.

## 1. Introduction

Microgravity induced skeletal muscle atrophy is well established in aerospace medicine. In 1999, it was first demonstrated that skeletal muscle cells were directly affected by space travel [1, 2]. However, the mechanism of muscle atrophy in astronauts lacks foundation other than microgravity-induced unloading. Skeletal muscle atrophy is characterized by reduced muscle fiber size and wasting. Skeletal muscle wasting is a predominate indicator of terminal illness because skeletal muscle is a homeostatic-sensitive organ. Any slight shift in balance causes skeletal muscle to dramatically reduce itself to maintain homeostasis. Currently, medical experiments on the International Space Station (ISS) have not revealed any substantial leads in how to prevent muscle atrophy or have investigated other reasons for muscle atrophy other than microgravity. This is described in one of NASA’s human research gaps M23: “Determine if factors other than unloading contribute to muscle atrophy during space flight” and lists potential risk factors as “inflammation, redox balance, energy balance, hydration, etc.” [3]. The potential risk factors in the NASA list are all associated with cholesterol and will be the focus of discussion in this article. Cholesterol is a fundamental and modifiable biochemical component in phospholipid bilayer. Hypercholesterolemia (HC) is characterized by high cholesterol in the blood with increased levels of low-density lipoproteins (LDLs) and low levels of high-density lipoproteins (HDLs). One study has demonstrated significant skeletal muscle fiber wasting as a result of ApoE inactivation and increased non-HDL cholesterol in LGMD2B mice. It was postulated that damaged vascular barriers allow the chronic leakage of plasma lipids into muscle tissue, resulting in muscle inflammation [4]. Microgravity shortened the body length and increased fat accumulation in microgravity-cultured worms compared to normal gravity cultured worms [5]. Impaired lipolysis in adipose tissues and skeletal muscles due to reduced mRNA expression levels of lipoprotein lipase (LPL) in adipose tissue and reduced LPL activity in skeletal muscle [6]. Impaired function of LPL in skeletal muscle mediates HC which ultimately leads to increased LDLs and muscle atrophy due to decreased blood supply caused by lumen obstruction [7]. This notion is supported by the fact that lower skeletal muscle mass index is associated with dyslipidemia and increased muscle mass is a must to prevent hypercholesterolemia [8]. These results suggest an association of skeletal muscle weakness with hypercholesterolemia and increased muscle mass and lowering LDLs as a therapeutic strategy to prevent muscle atrophy. Cholesterol (in the context of space) has become a significant health marker in heart disease, which NASA also aims to mitigate in astronauts [9]. However, the current collection of space-related medical research on cholesterol is insufficient and does not reveal much about the effect of microgravity on cholesterol. In fact, cholesterol is not a routine measurement for astronauts when landing [10]. According to NASA, hypercholesterolemia, obesity, diabetes, and hypertension could develop in astronauts [11]. Growing interest in hypercholesterolemia-induced muscle atrophy may provide novel insight to protect astronauts from such health risks [4, 12]. This review aims to critically evaluate the cholesterol-related microgravity research and emphasizes the detrimental consequences of undervalued cholesterol as a target in treating muscle atrophy in astronauts.

## 2. NASA-defined Spaceflight Health Risks

It is important to recognize that astronauts perform tasks inside and outside the International Space Station (ISS), and few researchers have considered that not all astronauts are tasked with spacewalks. Astronauts who participate in spacewalks could be potential outliers in medical experiments because of the physical impact from the severely outdated spacesuit. According to the audit of April 2017, only 11 of the original extravehicular mobility unit (EMU) spacesuits are still in use, 4 of which are available on the ISS and the rest are on Earth for maintenance [13]. This is prompted by the need to develop a next-generation spacesuit to address the limitations of the current spacesuit. In August 2021, NASA Office of Inspector General performed another audit to examine the current progress of the next-generation spacesuits for the ISS and Artemis missions [14]. This audit also revealed the health-related risks associated with the current spacesuit and examples of negative health outcome from specific missions. Spacesuit-related risks include decompression sickness, thermal regulation, shoulder injuries, hand injuries, malnutrition, and dehydration. In addition to this, astronauts are not completely protected by the current spacesuit from cosmic radiation during spacewalks or on the Moon. Compared to activities done safely inside the ISS, spacewalks are considered one of the most dangerous jobs in mankind and can certainly contribute to the physical and psychological stress of astronauts. However, similar health risks are associated within the ISS as described in Appendix B of NASA’s November 2021 audit [IG-22–005] [15]. Some notable mentions are injury due to the operations of extravehicular activity (EVA), altered immune response, inadequate food and nutrition, spaceflight-induced cardiovascular disease (CVD), and space radiation exposure. The same audit highlights how mitigation of human health risks requiring ISS microgravity testing will not be complete by 2030, the retirement date of the ISS, and 8 out the 12 critical human health risks will not be mitigated at an acceptable level for long-term spaceflight [15]. Although this schedule is based on an approximation, it can be delayed even further if research is not productive. This warrants critical evaluation of the current aerospace research efforts and what shortcomings they share to pinpoint the reason behind the slow progress.

## 3. Role of Cholesterol in Space Health Risks

**Figure 1:**
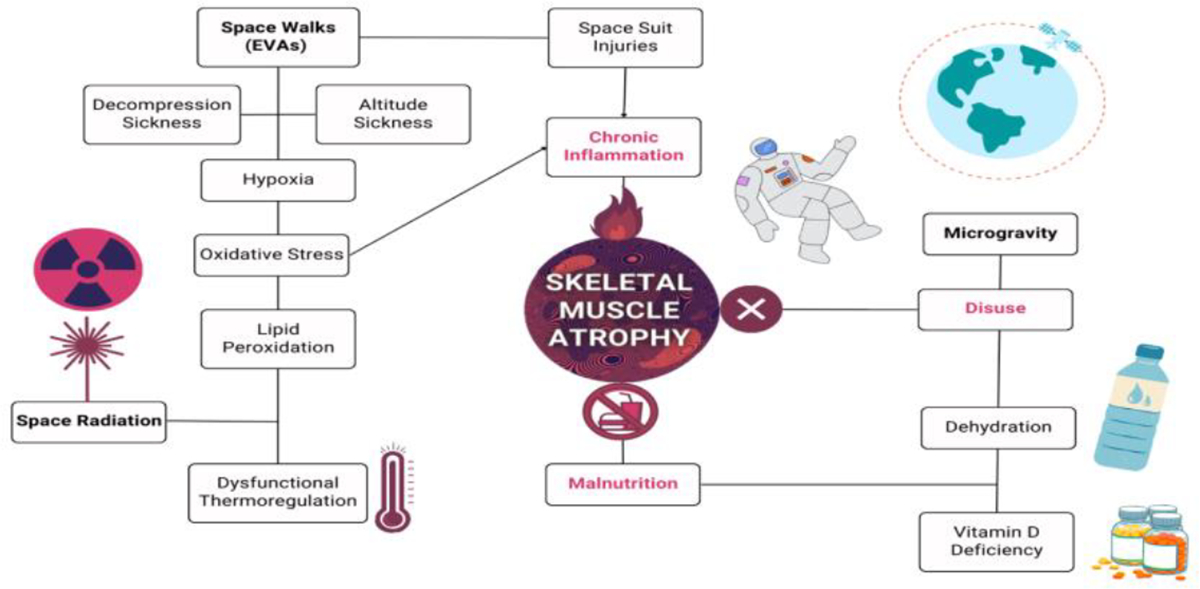
Spaceflight health risks that lead to skeletal muscle atrophy in astronauts. The terms highlighted in red are the known direct causes of muscle atrophy, which are disuse, malnutrition, and chronic inflammation. The bolded text represents the unique circumstances of spaceflight that astronauts are inevitably exposed. These include space walks, aka extravehicular activities (EVAs), space radiation, and microgravity. As illustrated, there are many intersecting confounding variables that may contribute to spaceflight-induced skeletal muscle atrophy. However, the one confounding variable that has yet to be considered is cholesterol, as it plays a role in almost all these clinical scenarios.

Microgravity is undoubtedly a main contender for muscle atrophy, but research in microgravity has an astronomical amount of confounding variables, leaving inconsistent and often uninterpretable results. The main concerns NASA described in their audits have one common variable: cholesterol. According to the history, cholesterol was first described as a perpetrator of heart disease in 1968, only 7 years after the first manned spaceflight in 1961 and around the same time as the Apollo moon landing in 1969. Whether or not the first biomedical researchers of NASA have considered these factors and how far to investigate them is up to debate. Since the discovery on the role of cholesterol role in heart disease, the recommended consumption of cholesterol is less than 300mg/d for astronauts, which has not changed since. The behavior of cholesterol in microgravity was demonstrated in a basic study where simulated microgravity enhanced lipid accumulation along imitation-vessels due to random particle direction [16]. In an environment of potent solar radiation, oxidation of cholesterol may exacerbate the health of astronauts. In each relevant health risk, cholesterol will be described in context of clinical scenarios that mimic health outcomes in microgravity (Figure 1).

## 4. Cholesterol and Decompression Sickness

Decompression sickness occurs when an astronaut’s body experiences sudden drop in surrounding pressure, such as their spacesuit. Decompression sickness is associated with terrestrial activities such as deep sea diving and non-pressurized aerial flying [17]. Hypercholesterolemia after high-fat meal increases risk for decompression sickness in divers [18]. Astronauts tend to eat before spacewalk to avoid eating in spacesuit. If preparing for an 8-hour spacewalk, it can be assumed that they are eating enough to sustain themselves for that period. Binge eating beforehand can spike serum cholesterol concentrations of an astronaut right before preparing for a spacewalk, as seen in diving. Decompression sickness is associated with oxygen toxicity because deep-sea divers and astronauts both breathe pure oxygen. However, it has long been known that concentrations of oxygen greater than normal breathing air has slow but detrimental effects. One of these effects include oxidative stress-induced inflammation and lipid peroxidation [19]. On the other hand, divers or astronauts returning from breathing pure oxygen results in decreased blood oxygen levels and results in hypoxia. This is also associated with high altitude sickness where there is a drop in oxygen pressure [20]. Acclimatization to high altitudes show significant positive correlation with increasing serum cholesterol [21]. High-altitude induced hypoxia are directly associated with increased non-HDL cholesterol [22]. Hypoxia can lead to chronic inflammation and oxidative stress, another common characteristic in astronauts, and will be discussed further in the following sections [23, 24].

## 5. Cholesterol and Thermoregulation

Astronauts have a water-cooling system in their spacesuits to help regulate their increased temperature during ISS spacewalks. Thermal regulation is challenging because astronauts may spend up to 8 hours in their spacesuits in direct solar radiation while also performing tasks. Cholesterol is a fundamental component of animal cell membrane homeostasis that can act as a buffer molecule to stabilize the phospholipid bilayer over a range of temperatures. Cholesterol lowers membrane fluidity in high temperatures and increases membrane fluidity in lower temperatures [25]. Astronauts experience increased core body temperature and are at risk for heat stress and hyperthermia [26]. On earth, increased ambient temperature is associated with increased levels of LDL and decreased levels in HDL [27]. Heat stress from a heat wave has shown to increase plasma cholesterol in a British population [28]. Hyperthermia is correlated with increased cellular cholesterol contents studied in mammalian cell lines [29]. Long-term heat exposure influenced cholesterol metabolism in pigs and temporarily increased serum and LDL cholesterol levels. However, inflammation or tissue damage was not present [30]. In hibernating brown bears, dyslipidemia and muscles were protected against lipid-specific oxidative damage due to higher plasma-antioxidants reserves [31]. Increased temperature may only be relevant to current space activities, whereas decreased temperatures on the Moon or Mars may pose a new threat in space exploration.

## 6. Cholesterol and Spacesuit Injuries

Extravehicular activity (EVA) injuries of hands and shoulders are usually sustained with the EMU spacesuit during spacewalks and EVA training. In zero gravity, astronauts tend to use their upper extremities for movement and physical tasks, versus their lower extremities, resulting in the overuse of shoulders and hands. The spacesuit hardware of the upper torso was the main cause of injury during active duty [32]. Although, shoulder injury has only increased recently in NASA perhaps due to changes in the space suit design or changes in the spaceflight requirements, such as all astronauts must be EVA certified in order to fly starting in 2000 [33]. Astronauts selected in the 1990’s have higher incidences in shoulder surgery. Astronauts who have performed more than five spacewalks were twice as likely to sustain shoulder injuries than astronauts who performed one spacewalk [34]. Shoulder injury is a broad term that encompasses many different types of myopathies or tendinopathies relating to the shoulder rotator cuff. Rotator cuff injury could manifest from hypercholesterolemia due to higher levels of total cholesterol, triglycerides, and LDL cholesterol [35]. Dyslipidemia and lipid deposition is associated with the failure of rotator cuff repairs and increased risk of retear [36]. NASA is fully aware of the detrimental effects of the spacesuit to cause shoulder injury in astronauts and have opened this issue for public collaboration. Texas Women’s University and Wichita State University are currently in the works for a mechanical sensor detection system to avoid shoulder injury [37, 38]. On the other hand, astronauts also experience handgrip fatigue due to pressurized gloves and limited mobility [39]. Those with smaller hands are more likely to sustain hand injuries, such as blisters, cuts, or joint pain [13]. From 1993 to 2010, NASA reports that 76 percent of astronauts sustained injuries of the fingernail, finger crotch, metacarpophalangeal joint, or fingertip [40]. Cholesterol deposits were found in extensor tendons of the hands in hypercholesterolemia patients [41, 42]. This could possibly increase the risk of astronauts developing hypercholesterolemia-induced arthritis [43]. Efforts are underway to improve pressurized gloves to mitigate hand injury, such as the next-generation high performance EVA gloves and the second-generation Space Suit Robot Glove (SSRG) [44, 45].

## 7. Cholesterol and Spaceflight Malnutrition

As mentioned before, astronauts may spend several hours performing an EVA without eating or drinking fluids. Single food bars were previously installed in EMU spacesuits, but were discontinued due to inconvenience (low calorie, extra weight, smearing on visor, limited storage space, etc.) [13, 46]. Astronauts prefer to eat a solid nutrient-dense meal before an EVA to remain satiated for as long as possible [47]. It is not clear if all astronauts are eating enough calories for their spacewalk or if astronauts over-eat to mitigate potential fatigue and hunger caused by the physical demands of an EVA. The food prepared for astronauts are heavily processed (freeze-dried) to meet sanitary and package requirements for long-term storage. Although fresh foods are more nutritional, they are prohibited due to shorter shelf-life and potential microbial contamination. Freeze-dried food and menu fatigue contribute to decreased appetite in astronauts [48]. Underconsumption of required nutritional intake may increase the risk of malnutrition in astronaut and subsequently increase oxidative stress in muscles. One study demonstrated that depriving nutrients in C2C12 muscle cells significantly increased the levels of reactive oxygen species (ROS) [49]. Food prepared in the ISS are higher in cholesterol (300 mg as of year 2020) than the standard recommendation of 200 mg [50]. If astronauts eat big meals, this may suddenly cause serum cholesterol to spike, such as consuming high amounts of sugar or eggs [51, 52]. It would be beneficial to study the body metabolism of astronauts before and after an EVA to investigate metabolic efficiency. Because most seasoned astronauts are middled aged, they are at higher risk for cardiovascular disease. Age-related metabolic alterations may affect how nutritional intake impacts endocrine functions, muscle mass homeostasis, and lipid profile [53]. Aging is correlated with dyslipidemia and cardiovascular disease [54, 55]. Aging also impacts muscle mass through sarcopenia and chronic inflammation [56, 57]. Striated muscle atrophy has been observed in cardiovascular disease [58]. Cardiovascular disease risk may be due to hypercholesterolemia. High LDL-cholesterol has been associated with cardiovascular mortality and lowering LDL-C levels are beneficial for preventing cardiovascular disease in men [59, 60]. Moreover, high levels of oxidized-low density lipoprotein (ox-LDL) exacerbate the progression of atherosclerosis [61]. Although NASA aims to mitigate cardiovascular disease in astronauts, it is concerning that cholesterol is not routinely measured, which leads to the lack of cholesterol-specific research [10].

On the other spectrum, decreased vitamin D was the most striking nutritional changes during spaceflight. Vitamin D shares the same precursor as cholesterol, 7-dehydrocholesterol (7-DHC) [62]. UV light is completely shielded on the ISS and spacesuits have built in UV-blocking material. Therefore astronauts are unable to endogenously synthesize vitamin D for long period of time. Chronic vitamin D deficiency is a critical concern for long-term space exploration because of the decreased calcium absorption and subsequent bone wasting [63]. Dyslipidemia, increased total cholesterol (TC), low-density lipoprotein cholesterol (LDL-C), decreased in high-density lipoprotein cholesterol (HDL-C) levels can manifest from vitamin D deficiency as well [64]. Astronauts also experience prolonged states of dehydration both in the ISS and during EVA spacewalks. Dehydration occurs after microgravity-induced fluid shift during spaceflight [65]. The recommended daily intake of water for astronauts is eight ounces per hour, but the disposable in-suit drink bag only contains 32 ounces of water, only sufficient for 4 hour spacewalks [66]. The average period of an EVA spacewalk in 2021–2022 is about 7–8 hours, which suggests that one drink bag is not enough for the entire mission [67]. Dehydration can lead to hypertension, muscle fatigue, and dizziness [68–70]. Cholesterol and hydration play a critical role in cell membrane fluidity and are directly correlated with each other [71]. This is demonstrated by increased serum cholesterol concentration in blood tests in dehydrated patients [72]. Therefore, it begs the question of whether the reported cholesterol levels in all previous human astronaut research is consistent or not.

## 8. Cholesterol and Space Radiation

In addition to UV radiation, astronauts experience high exposure to space radiation. Space radiation, or cosmic radiation, consists of high-charged energy particles, X-rays, and gamma rays. It is known that lipid peroxidation produced from space radiation [73]. LDL-cholesterol in particular is a vulnerable target of radiation-mediated oxidation [74]. Six hour exposure to UV radiation causes cholesterol to increase by 21.6-fold and direct cell membrane modification [75]. The effects of long-term exposure to dangerous levels of gamma radiation were significantly associated with hypercholesterolemia in Japanese atomic bomb survivors [76, 77]. For these reasons, cardiovascular disease and cancer are negative outcomes of space radiation that need to be mitigated before considering deep-space exploration [78].

## 9. Cholesterol and Space-induced Altered Immune Response

Astronauts experienced allergy-like symptoms, such as prolonged congestion, rhinitis, sneezing, and skin rashes, while traveling in space for long periods of time, which prompted investigation of how microgravity affects immune response [79]. Chronic inflammation was observed in astronauts due to many confounding factors, such as oxidative stress, but the mechanism behind microgravity-induced immune hyperactivity is not clear [80]. Since oxidative stress is a major contender of space risk, oxidation of LDL-cholesterol should not be ignored as a predictor of chronic inflammation. Hypercholesterolemia is often characterized by chronic inflammation response to LDL-cholesterol dominated macrophages, or foam cells [81]. Macrophages studies in microgravity are very diverse, making it more difficult to come to a significant conclusion [82]. Hyperlipidemia enhances neutrophil activity and are also elevated during microgravity as a response to oxidative stress [83, 84]. Response to dendritic cells diminished in microgravity as demonstrated by the suppression of T-cell reactivity [85, 86]. Microgravity studies have also shown hypoxia-inducible factor 1α (HIF-1α) as a potential therapeutic target for its role in macrophage and T-cell activation [87].

## 10. Cholesterol and Muscle Atrophy in Astronauts

As mentioned above, NASA lists potentially causative factors for space-induced skeletal muscle atrophy, including inflammation, redox balance, energy balance, hydration, and others [3]. Evidence provided so far support the role of cholesterol in all these potential causes/factors of muscle atrophy. Understanding cholesterol on its very basic level is extremely important to its homeostatic functions in cell anatomy. Too much cholesterol can cause negative effects on important skeletal muscle membrane-protein species, such as those found in the transverse tubule (t-tubule). The t-tubule is considerably more cholesterol-rich than the sarcolemma and increasing this level further impedes the trafficking of intracellular glucose transporter, GLUT4, to the t-tubule and other surface membranes [88, 89]. Increased t-tubule cholesterol concentration may also may negatively alter voltage-gated Ca^2+^ channels [90]. In microgravity (where cholesterol may increase through many variables), astronauts may experience diabetic symptoms and dysfunctional metabolism, rendering carbohydrate/glucose intake as potential waste. Depletion of this cholesterol impairs excitation and contraction coupling. [91] In addition, high cholesterol causes inhibition of ATPases by overloading cholesterol in the striated muscle sarcolemma [92, 93]. Cholesterol depletion induced by statin drugs, used by some astronauts, may exacerbate muscle atrophy [94]. Astronauts who need rotator cuff surgery may be at risk for poor repair outcomes due to fatty infiltration and rotator cuff muscle atrophy [95]. Re-analysis of the twin study landing data show that sharp increase of cytokines and chemokines, and other inflammatory markers, such as IL-6, IL-10, IL-1β, IL-1Ra, CRP, CCL2, and TNF-α, suggest regenerative response to atrophy rather than inflammatory response [96]. In fact, chronic inflammation can disrupt proteins in the skeletal muscle fiber triggering atrophy [97]. It was demonstrated in rats flown in space for 12.5 days that muscle atrophy occurred and it was characterized by the dysfunctional microcirculation, denervation, infiltration and phagocytosis of cellular debris by macrophages and neutrophils in necrotized skeletal muscle fibers [98]. Chronic inflammation in skeletal muscle is dependent on macrophage kinetics and disturbance in cell signaling can lead to muscle fiber degeneration [99]. In chronic inflammation, both M1 and M2 macrophages increase and compete for arginine metabolism as it is a shared substrate for iNOS and arginase. However, M2c macrophages reduce the activity of M1 macrophages, leading to a shift in metabolism from iNOS to arginase, resulting in a pro-fibrotic environment. M2 macrophages also increase myogenic factors, such as MYOG and MYOD [100].

Inflammation-induced striated muscle atrophy has been observed in the dysregulation of muscle fibers such as actin, myosin, and titin [101–103]. Actin fragments were generated by caspase-3 and further degraded by a ubiquitin-proteasome [104]. Muscle-specific E3 ubiquitin-ligase, TRIM63 (or MuRF1), has been shown to degrade myosin light chains 1 and 2, myosin heavy chains, and myosin-binding protein C [105]. TRIM63 has also been observed in titin degradation in cardiomyopathies [106]. In hindlimb unloading studies, proteolytic titin fragments increased significantly after 7-days of gravitational unloading [107]. Current spaceflight-related muscle research suggests that studies should focus on catabolic state markers, such as FOXO1 and muscle-specific TRIM63 (MuRF1) [108]. However, a recent transcriptome study done in 2021 has shown that deletion of MuRF1 in mice did not prevent muscle atrophy during spaceflight and suggested Cacng1 as a new target for microgravity-induced atrophy. The study also demonstrated that transfecting myotubes with active mutant of FOXO3, FOXO3a, decreased average myotube diameter by 27.5% [109, 110]. Mitochondrial function and mitochondrial DNA (mtDNA) can also be another relevant target for microgravity-mediated muscle atrophy caused by oxidative stress, DNA damage, and inflammation [111, 112]. Reactive oxygen species (ROS) can activate NF-κB pathways and subsequent release of cytokines like TNF-α, as well as damaging the sarcolemma and contractile proteins, exacerbating muscle dysfunction in dystrophic muscle cells[49]. The plasticity of skeletal muscle is often challenged by hypercholesterolemia and dyslipidemia. Skeletal muscle can fluctuate between hypertrophy, as seen in bodybuilders, and atrophy, as seen with disuse. Lipotoxicity can trigger inflammation and tissue damage as pro-inflammatory cytokines and adipokines are released from resident macrophages and adipocytes [113]. Increased cholesterol levels have been shown to intensify muscle wasting in the Duchenne muscular dystrophy (DMD) mouse model [114]. This suggests that hypercholesterolemia, through inflammation and fatty infiltration, can promote skeletal muscle atrophy. Age-related muscle atrophy, sarcopenia, reveal how low muscle mass and muscle strength are risk factors for disability and mortality [57]. Research on sarcopenia has suggested OPA1 as a potential therapeutic target for age-related muscle loss and chronic inflammation. Skeletal muscle sacrifices its own reserves to regulate metabolic dysfunction in lipid and glucose homeostasis in any state of health, which is why skeletal muscle atrophy is often observed in a range of diseases, such as infections to cancer [115]. Altered lipid metabolism plays a role in skeletal muscle weakness, as shown in obesity-like disease models. In rats, short-term high fat diet impaired function in oxidative-type skeletal muscles [116]. For patients with spinal muscular atrophy (SMA), malnutrition is a major concern because of defects in fatty acid transport and mitochondrial β-oxidation [117, 118]. In addition to altered lipid metabolism, glucose metabolism alteration may cause skeletal muscle atrophy, as observed in diabetes [119]. Glucose is essential for skeletal muscle function because it is responsible for maintaining blood glucose homeostasis by metabolizing 80% of consumed glucose. It has been shown in mice, knock out for skeletal-muscle-specific endogenous circadian clock, Bmal1, leads to glucose metabolism and systemic glucose homeostasis disruption [120]. A metabolic organ such as skeletal muscle is sensitive to homeostatic disturbances and may lead to imbalances of protein synthesis and degradation. Therefore, insulin-resistance may trigger skeletal muscle protein degradation through mTOR complex 1 signaling pathway (mTORC1) [121]. Hyperglycemia may also trigger muscle atrophy by WWP1/KLF15 pathway in diabetes [122]. Metabolic reprogramming may induce muscle mass wasting through impaired skeletal muscle specific mitochondrial pyruvate carrier (MPC), and upregulating the hepatic gluconeogenesis in the Cory cycle and fatty acid oxidation [123]. It has been hypothesized that microgravity-mediated chronic inflammation can develop into dysmetabolic conditions that include imbalance of lipid and glucose metabolism [124].

## 11. Future Prospective and Potential Targets

**Figure 2:**
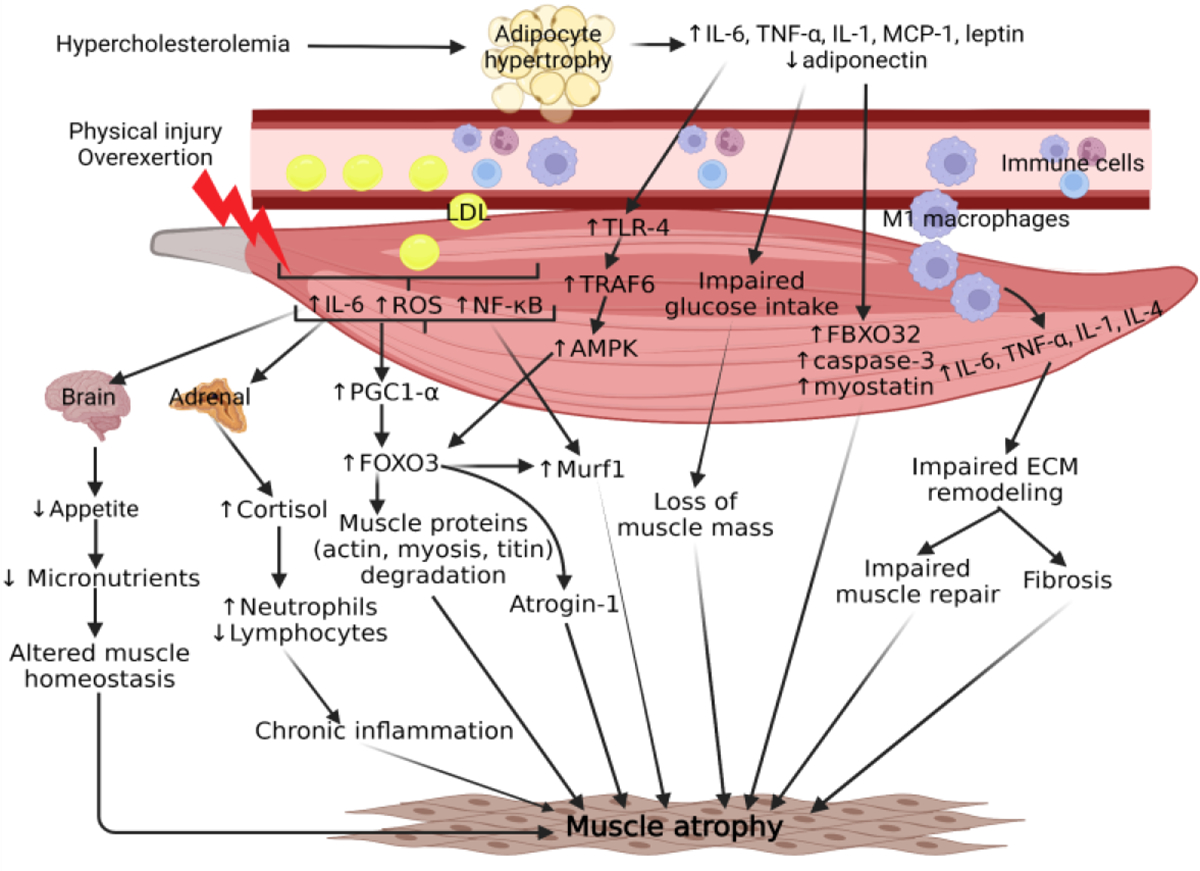
Chronic inflammation is strongly implicated in molecular mechanisms contributing to hypercholesterolemia-mediated skeletal muscle atrophy. The result of high cholesterol can increase adipocyte size and recruitment of immune cells, notably pro-inflammatory macrophages, into the peripheral skeletal muscle tissue. Both adipocytes and infiltrating macrophages contribute to a chronic signaling cascade of cytokines that ultimately lead to upregulation of fibrosis and atrogenes, such as MuRF1 and Atrogin-1. Physical injury or overexertion may further exacerbate chronic inflammation by inducing the release of muscle-specific cytokines, resulting in elevated cortisol levels from the adrenal gland and decreased appetite from the hypothalamus. This persistent crosstalk disrupts skeletal muscle homeostasis and contributes to muscle atrophy. Skeletal muscle is a metabolically sensitive organ and relies on the efficiency of circulating nutrients to maintain muscle mass. High concentrations of cholesterol in the blood have been demonstrated to cause muscle atrophy in peripheral artery disease due to poor circulation from plaque formation in the femoral arteries. Further investigation is needed to thoroughly understand the extent to which cholesterol plays a role in muscle atrophy.

The main factors precipitating space-induced skeletal muscle atrophy are cholesterol and chronic inflammation (Figure 2). Based on the scientific literature available about inflammatory mediators, oxidative stress, hypercholesterolemia, altered metabolism, and muscular protein degradation, there is an obvious link between muscle atrophy and cholesterol. However, it is gravely important to point out the potential contraindications of implementing statins as a treatment for hypercholesterolemia because statins may also cause myopathies, such as myalgia and rhabdomyolysis [125, 126]. Additionally, glucocorticoids should not be considered as a potential therapy for chronic-inflammation-induced muscle atrophy because it increases the rate of the ubiquitin-proteasome system [127]. Hypercholesterolemic LGMD2B mice with muscle atrophy have developed fatty infiltration and inflammation in limb and girdle muscles histology, which suggests chronic cytokine release from adipocytes and macrophages [4]. Macrophage cytokine overexpression of IL-6, TNF-α, and IL-1 have been demonstrated to increase myogenic factors and FOXO transcription factors leading to the subsequent increase titin degradation through MuRF1 (TRIM63) [99, 128, 129]. With the acknowledgment of microgravity-induced atrophy occurring even with MuRF1 deletion in mice, the mechanism between hypercholesterolemia and chronic inflammation in skeletal muscle must be investigated further to elucidate potential therapeutic targets. Studies investigating the interaction between skeletal muscle cytokines, also known as myokines, and other key organs, such as adipose tissue and the brain, have highlighted the multifaceted role that upregulated levels of IL-6 play in chronic inflammation and the decrease in appetite after exercise. Depending on the signaling origin, such as canonical myeloid cells or non-canonical adipocytes and muscle, IL-6 could play an pro-inflammatory or anti-inflammatory role in skeletal muscle, respectively [130]. However, IL-6 is a gravity-sensitive myokine and may contribute to homeostatic dysregulation of muscle [131]. Elucidating the mechanisms of IL-6 and other myokines in microgravity would provide potential targets for developing anti-inflammatory therapies for astronauts. Space travel leads to an increase in production of reactive oxygen species (ROS), which causes cellular stress and damage to astronauts, including muscle atrophy.

The development of an antioxidant cocktail was proposed to maintain the health of astronauts and requires consideration of factors such as the physiological effects of ROS on the body, genetic predisposition of astronauts to damage, and the efficacy of antioxidants. [132]. For ROS-related muscle atrophy, it was demonstrated that two antioxidants, N-acetyl-L-cysteine (NAC) and pyrroloquinoline quinone (PQQ), significantly decreased the development of skeletal muscle atrophy induced by fasting [49]. Exposure to the microgravity and radiation leads to destroyed red blood cells and excessive iron stores, which results in an imbalance in redox homeostasis, leading to oxidative damage of cells and injuries in the musculoskeletal system. Therefore, antioxidants and exogenous iron chelators were suggested as potential therapies [133]. An article reviewed the significance of certain nutrients in combating the harm brought about by microgravity during space missions. To mitigate oxidative stress, it was suggested to increase the consumption of antioxidants such as vitamins A, C, and E, omega-3 fatty acids, and minerals like copper, zinc, manganese, selenium, and iron through the diet. The combination of dietary defenses and the production of endogenous antioxidants could potentially play a significant role in guarding against oxidative damage. To address cardiovascular problems, it was advised to follow low-glycemic index diets. Vitamin D_3_ was deemed important for preventing bone damage, but high doses of it could lead to hypercalcemia, kidney stones, and the calcification of soft tissues [134]. The TCA cycle could be another target for microgravity-induced atrophy as all participating enzymes were reported to have low gene and protein expression, such as citrate synthase, aconitase, isocitrate dehydrogenase, succinate dehydrogenase, fumarase, and malate dehydrogenase [5]. In muscle atrophy, there is a reduction in energy production, which leads to decreased muscle function. By targeting the TCA cycle, it may be possible to increase energy production in the muscle and thus, counteract or prevent muscle wasting. Metabolomics and epigenomics are promising bioinformatic studies that focus on the shifts in environments and how that impacts gene expression. Fundamental areas of space biology research include oxidative stress, DNA damage, mitochondrial dysregulation, epigenetics, telomere length alterations, and microbiome shifts [135]. The Gene Lab database of NASA is useful for researchers interested in bioinformatic analyses of experiments performed in space. However, it limits human data due to privacy concerns and access must be authorized by the NASA Human Research Program. Processing of the bioinformatic data also takes time and requires interdisciplinary understanding of multiple statistical methods.

## 12. Conclusions

The area of hyperlipidemia-associated muscle atrophy in astronauts is important but is poorly investigated. Skeletal muscle and cholesterol share an evolutionary-conserved function in energy metabolism and storage. Overall, this review critically evaluated the efforts of NASA, or lack thereof, in the coordination of their life sciences departments. This was also highlighted in the October 2015 audit of NASA [136]. A critical description is also presented as to how the twin study is an example of the small sample size that does not represent the population of astronauts and could lead to vague conclusions about the effects of spaceflight. The basis of scientific research requires larger population and decrease the workload off the few astronauts who are in high demand. Despite the recent renewal of TRISH (Translational Research Institute of Space Health) by the NASA Human Research Program in December 2020, more biomedical researchers and physicians with aerospace background are needed to facilitate meaningful investigations. Aerospace medical programs need to be marketed as much as aerospace engineering programs. Besides military medical residencies, only one civilian residency program for aerospace medicine exists at the University of Texas Medical Branch (UTMB) and one civilian fellowship program at the Mayo Clinic [137]. Recently, UCLA medical school opened their new aerospace medicine fellowship program starting July 2022 [138]. Whether cholesterol levels increase or decrease due to microgravity, better protocols and experimental methods must be established to thoroughly investigate the role of cholesterol in astronaut health. Because cholesterol is an important biomarker for many co-morbidities of cardiovascular disease, it cannot be ignored in aerospace biomedical research.

## Data Availability

Not applicable since the information is gathered from published articles.
